# Tetrafunctional Block Copolymers Promote Lung Gene Transfer in Newborn Piglets

**DOI:** 10.1016/j.omtn.2019.02.016

**Published:** 2019-02-26

**Authors:** Ignacio Caballero, Mickaël Riou, Océane Hacquin, Claire Chevaleyre, Céline Barc, Jérémy Pezant, Anne Pinard, Julien Fassy, Roger Rezzonico, Bernard Mari, Nathalie Heuzé-Vourc’h, Bruno Pitard, Georges Vassaux

**Affiliations:** 1 INRA Centre Val de Loire – Université de Tours, UMR-1282 Infectiologie et Santé Publique (ISP), 37380 Nouzilly, France; 2 INRA Centre Val de Loire, UE-1277 Plateforme d’Infectiologie expérimentale (PFIE), 37380 Nouzilly, France; 3 Université Côte d’Azur, INSERM, CNRS, IPMC, Valbonne, France; 4 FHU-OncoAge, Nice, France; 5 Centre d’Etude des Pathologies Respiratoires, U1100, INSERM, Tours, France; 6 CRCINA, INSERM, Université d’Angers, Université de Nantes, Nantes, France

**Keywords:** tetrafunctional block copolymers, lungs, gene therapy, non-viral vector, newborn pigs

## Abstract

Tetrafunctional block copolymers are molecules capable of complexing DNA. Although ineffective *in vitro*, studies in mice have shown that the tetrafunctional block copolymer 704 is a more efficient lung gene transfer agent than the cationic liposome GL67A, previously used in a phase II clinical trial in cystic fibrosis patients. In the present study, we compared the gene transfer capacity of the 704-DNA formulation and a cationic liposome-DNA formulation equivalent to GL67A in a larger-animal model, the newborn piglet. Our results indicate an efficacy of the 704-DNA formulation well above one order of magnitude higher than that of the cationic liposome-DNA formulation, with no elevated levels of interleukin-6 (IL-6), taken as a marker of inflammation. Transgene expression was heterogeneous within lung lobes, with expression levels that were below the detection threshold in some samples, while high in other samples. This heterogeneity is likely to be due to the bolus injection procedure as well as to the small volume of injection. The present study highlights the potential of tetrafunctional block copolymers as non-viral vectors for lung gene therapy.

## Graphical Abstract

**Figure:**
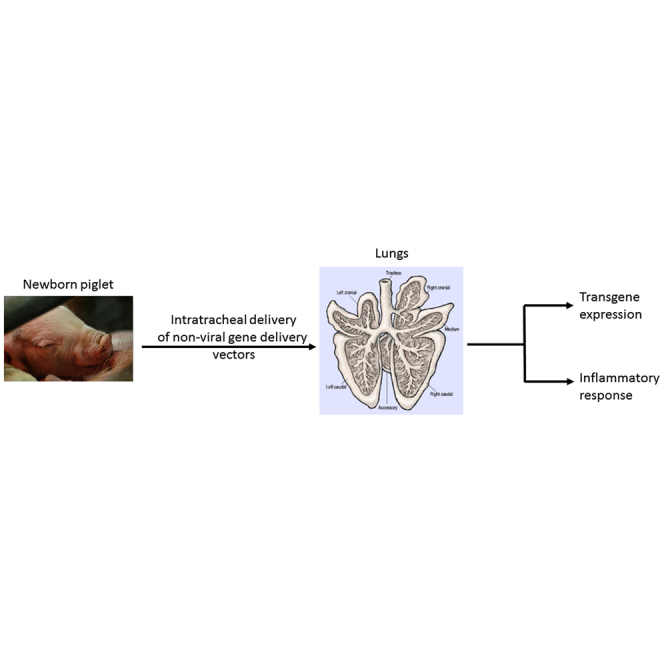

## Introduction

Lung gene transfer has potential application in a large range of pathologies. These include inherited diseases such as cystic fibrosis (CF)^1^ and pathologies with mixed origins, such as asthma and chronic obstructive pulmonary disease.^1^ Several acquired diseases that lack satisfactory treatments, such as primary lung cancers or metastases of distant cancers and idiopathic pulmonary fibrosis, could also be considered as indications for gene therapy.^1^ More surprisingly, gene transfer to the lungs has been proposed as a method to produce proteins for release into the circulation.^2^, ^3^, ^4^

To achieve sufficient gene delivery, the development of vectors relevant to the pathology and to the expected therapeutic schedule is central. Viral vectors are highly efficient but their utilization has been hampered by their immunogenic-proinflammatory properties,^5^ which render them inappropriate for applications in pathologies where the lungs are severely inflamed and/or that require repeated administrations. Recent developments have led to the emergence of integrating pseudotyped lentiviruses.^6^ These can promote long-lasting transgene expression in rodent models,^7^, ^8^, ^9^, ^10^, ^11^ and their relevance to the clinical situation is currently under investigation.^10^, ^12^

Non-viral gene delivery vectors represent an attractive alternative. These are synthetic, chemically defined organic molecules complexed with DNA. They are less likely to induce a strong inflammatory response and are particularly relevant in indications that require transient gene expression and/or repeated administrations. Within this field, the most studied reagent is the cationic lipid formulation, GL67A. This formulation has been shown to transfect mouse and ovine lungs *in vivo*^13^, ^14^, ^15^ and to be suitable for repeated administration.^14^, ^15^ GL67A has been administered successfully to healthy volunteers and to CF patients.^16^, ^17^ Repeated administration of GL67A to CF patients resulted in stabilization of the disease in a randomized, double-blind, placebo-controlled, phase 2b trial.^18^

Formulations composed of DNA and non-ionic amphiphilic block copolymer have been reported to successfully transfect skeletal and cardiac muscles.^19^, ^20^, ^21^, ^22^ Intratracheal delivery of a similar formulation led to a gene transfer level equivalent to that promoted by a polyethylenimine-based formulation, but with reduced inflammation.^23^ Tetrafunctional block copolymers have a tetrafunctional structure consisting of four poly(ethylene oxide) -poly(propylene oxide) blocks centered on an ethylenediamine moiety. They form small complexes with DNA,^24^ and their potential as vectors for cardiac and muscle gene transfer has been described.^20^, ^24^, ^25^, ^26^ The ability of tetrafunctional block copolymers to deliver DNA in muscles for expression of genes of therapeutic interest has been demonstrated in mouse models of hepatocellular carcinoma,^27^ allergic asthma,^28^, ^29^ and colorectal cancer,^30^ against the CF *Mycobacterium abscessus*,^31^, ^32^ against transposase-derived proteins encoded by human neogenes,^33^ and against Zika virus.^34^ In an attempt to identify more efficient non-viral formulations for lung gene transfer, we used molecular imaging to screen and assess *in vivo*, in mice, the transfection potential of a range of tetrafunctional block copolymers, and we identified the tetrafunctional block copolymer 704 as an efficient non-viral vector.^35^ DNA/704 formulation-mediated lung gene transfer resulted in higher levels of reporter gene expression than the cationic liposomal GL67A formulation used in the UK CF clinical trial on CF patients.^18^ The inflammatory response associated with this gene transfer was lower than that induced by the GL67A formulation, and the 704 formulation was amenable to repeated administrations. The 704 formulation was also shown to allow lung expression of Fractalkine-Fc.^36^ This expression reduced lung metastasis.^36^ These results emphasized the relevance of the 704 formulation as a non-viral gene delivery vector for lung gene therapy. The aim of the present study was to determine the applicability of this formulation in larger-animal models. We therefore compared, in newborn piglets, lung gene transfer achieved using either a DNA/liposome formulation similar to GL67A or the DNA/704 formulation.

## Results

### Clinical Follow-Up and Hematological Analyses in Newborn Piglets after Administration of the Formulations

No adverse clinical events were observed upon administration of the non-viral formulations. No hyperthermia, tachycardia, prostration, anorexia, or weight loss were observed in newborn piglets. Red blood cell parameters were normal for piglets (Table 1). There were no clear differences in the other clinical and biological parameters evaluated in all animals, except for the percentage of lymphocytes (Table 1). The increase in lymphocyte number and the lymphocytes/neutrophils (L/N) ratio at 48 h (Table 1) was observed in most piglets and may have been the result of the effect of the anesthesia and inoculation of liquid in the lungs, although such an increase has been documented during the development of porcine immunity of the newborn piglet.^37^, ^38^

**Table 1:** Clinical Parameters in Newborn Piglets after Intratracheal Administration of the Non-viral Vectors

	Controls (n = 2)		704 1 mg DNA (n = 2)		Liposome 1 mg DNA (n = 2)		704 0.5 mg DNA (n = 1)		704 1 mg DNA (n = 1)	
Time point (h)	0	48	0	48	0	48	0	48	0	24
Temperature (°C)	37.8 ± 0.1	37.8 ± 0.4	38.1 ± 0.4	38.2 ± 0.4	37.7 ± 0.3	37.5 ± 1.0	37.6	36.9	37.4	36.4
Heart rate (bpm)	102^a^ ± 110	28.5 ± 8	34.5 ± 5	46.5 ± 4	108.5^a^ ± 94	47 ± 21	64	103	169^a^	149
White blood cells (m/mm^3^)	7.2 ± 3.0	5.8 ± 0.4	7.4 ± 2.9	6.3 ± 0.3	5.5 ± 0.0	4.3 ± 0.4	7.2	6.9	6.2	4.2
Lymphocytes (%)	41.2	74.6	65.4	76.0	49.6	76.05	62.0	63.3	52.3	76.1
Neutrophils (%)	26.9	13.2	17.9	11.2	29.3	11.2	20.9	21.4	30.4	30.1
Ratio L/N^b^	1.51 ± 0.3	11.7 ± 12.7	3.7 ± 0.7	9 ± 6.6	4.3 ± 0.7	6.9 ± 0.4	2.96	2.65	1.7	1.8
Red blood cells (M/mm^3^)	4.3 ± 0.4	4.4 ± 0.1	4.8 ± 0.9	4.9 ± 0.4	4.6 ± 0.4	3.9 ± 0.3	4.5	4.7	4.9	5.7
Hemoglobin (g/dL)	9.4 ± 0.6	9.9 ± 0.7	15.5 ± 7.7	9.3 ± 0.3	10.0 ± 0.0	7.6 ± 0.6	10.6	10.4	11.1	7.4
Hematocrit (%)	28.9	28.4	30.5	31.5	27.8	27.7	32.9	31.1	31.2	38.3
Platelets (m/mm^3^)	241 ± 6	303 ± 177	235 ± 2	303 ± 69	206 ± 49	366 ± 26	164	360	194	164

a Induction of stress during restraint inducing an increase in heart rate.

b Ratio L/N, ratio lymphocytes/neutrophils.

### Liposome-Mediated Lung Gene Transfer

**Figure 1:**
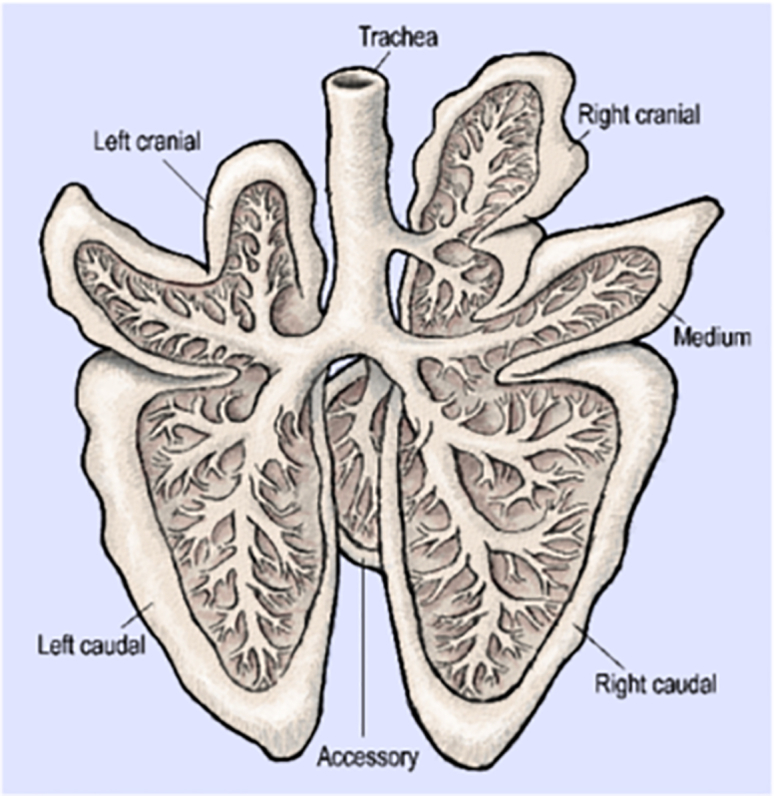
Anatomical Representation of Pig Lungs The trachea and the different lobes of the lungs are represented.

**Figure 2:**
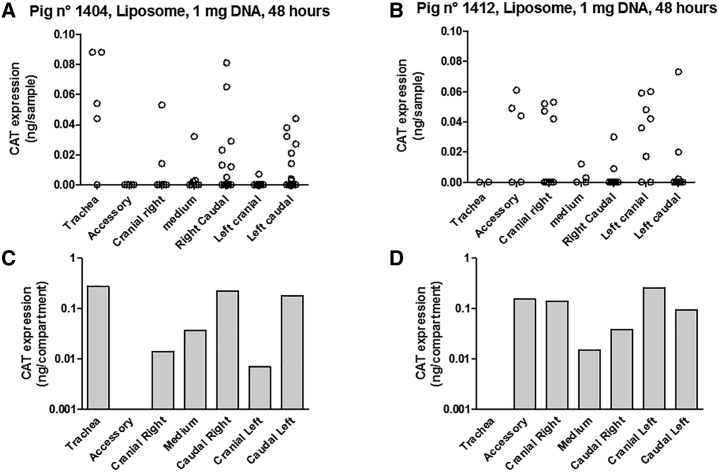
Liposomal Gene Transfer to Newborn Pig Lungs CholP/DOPE/DMPE-PEG5000 liposome formulation was used to deliver 1 mg plasmid. Forty-eight hours later, the animals were euthanized, lungs were cut into small cubes, and CAT activity was measured in individual cubes. (A and B) Transgene expression in individual cubes and the repartition in the different lung compartments. The total amount of CAT protein measured in each lung compartment of pig n° 1404 (A) and pig n° 1412 (B) are presented in (C) and (D), respectively.

An anatomical representation of the different lobes of pig lungs is presented in Figure 1. We first performed gene transfer experiments using a liposomal composition mimicking GL67A liposomes, consisting of a cationic cholesterol associated with the same neutral lipid and poly ethylene glycol (PEG) lipids that are present in GL67A, i.e., paromomycine-derived cholesterol/dioleyl phosphatidyl ethanolamine/dimyristoyl phosphatidyl ethanolamine-PEG (CholP/DOPE/DMPE-PEG5000). Intratracheal administration of the liposome-DNA complex was used to deliver 1 mg plasmid encoding the chloramphenicol acetyl transferase (CAT) coding sequence. Forty-eight hours later, the animals (n = 2) were euthanized, lungs were collected and sampled, and transgene expression was measured. Figures 2A and 2B show that transgene expression was detected in the lungs of both animals. Overall, gene transfer was observed in all lung compartments, although inter-individual differences were observed. The accessory lobe and the trachea were devoid of CAT expression in animal n^o^ 1404 and n^o^ 1412, respectively. Within a particular compartment, gene transfer was heterogeneous, with some samples in which CAT expression was below the detection threshold while other samples presented elevated CAT expression. To obtain a quantitative assessment of the level of gene transfer, summation of the amounts of CAT protein (in ng) in individual samples was performed in all the lung compartments. The data presented in Figures 2C and 2D show that intratracheal administration of 1 mg CAT-expression plasmid formulated with the liposome CholP/DOPE/DMPE-PEG5000 resulted in a total level of CAT protein expression ranging from 0.01 to 0.1 ng in the different lung compartments, 48 h later (Figures 2C and 2D). Finally, the total amount of CAT protein produced in the lungs upon liposomal gene transfer was 0.743 ng for pig n° 1404 and 0.706 ng for pig n° 1412.

### Tetrafunctional Block Copolymer-Mediated Lung Gene Transfer

**Figure 3:**
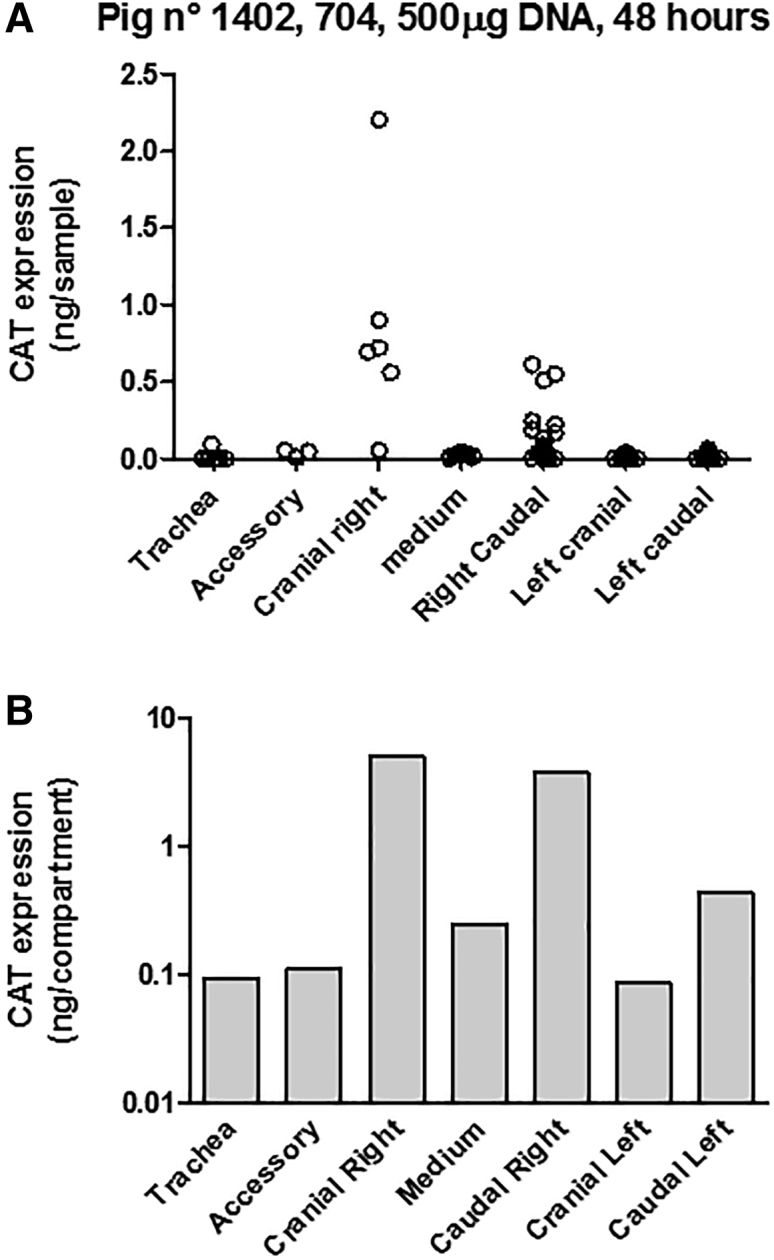
Tetrafunctional Block Copolymer-Mediated Gene Delivery of 0.5 mg Plasmid The 704-DNA formulation was used to deliver 0.5 mg plasmid. Forty-eight hours later, the animals were euthanized, lungs were cut into small cubes, and CAT activity was measured in individual cubes. (A) Transgene expression in individual cubes and the repartition in the different lung compartments. The total amount of CAT protein measured in each lung compartment of pig n° 1402 (A) is presented in (B).

**Figure 4:**
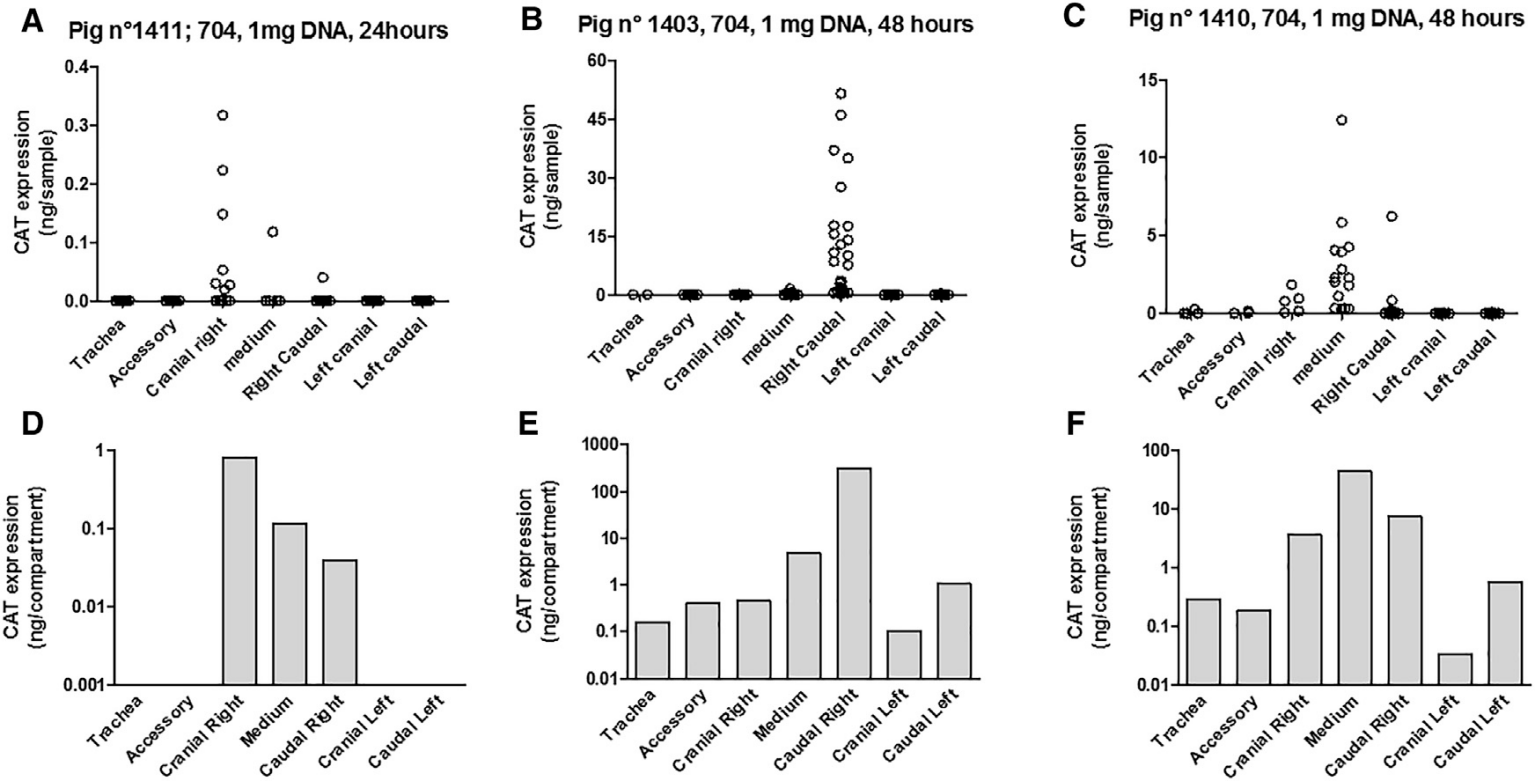
Tetrafunctional Block Copolymer-Mediated Gene Delivery of 1 mg Plasmid The 704-DNA formulation was used to deliver 1 mg plasmid. Twenty-four hours (A and D) or forty-eight hours (B, C, E, and F) later, the animals were euthanized, lungs were cut in small cubes, and CAT activity was measured in individual cubes. (A–C) Transgene expression in individual cubes and the repartition in the different lung compartments. The total amount of CAT protein measured in each lung compartment of pig n° 1411 (A), pig n° 1403 (B), and pig n° 1410 (C) are presented in (D), (E), and (F), respectively.

**Figure 5:**
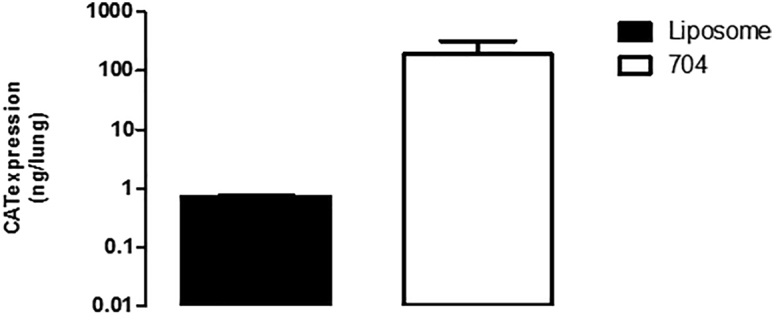
Comparison of the Total Amount of CAT Protein Produced in the Whole Lung of Liposome- and Tetrafunctional Block Copolymer-Mediated Gene Delivery of 1 mg Plasmid The liposomal-DNA or 704-DNA formulation was used to deliver 1 mg plasmid. Forty-eight hours later, the animals were euthanized, lungs were cut into small cubes, and CAT activity was measured in individual cubes. CAT protein amounts in the whole lungs of newborn pigs were added. The data represent the mean ± SEM of n = 2.

A similar set of experiments was performed using the tetrafunctional block copolymer formulation to deliver 0.5 mg (Figure 3) or 1 mg (Figure 4) CAT-expression plasmid. As for liposomal gene transfer, the pattern of CAT protein expression was heterogeneous within lung compartments, with some individual samples containing no detectable CAT protein while others exhibited a high level of CAT protein. In pig n° 1411, analysis of transgene expression performed 24 h after administration of the formulation containing 1 mg plasmid DNA showed a lack of transgene expression in the trachea and the left cranial, left caudal, and accessory lobes (Figures 4A and 4D). By contrast, transgene expression was detectable in all lung compartments after 48 h (pig n° 1402, Figure 3; and pig n° 1403 and n° 1410, Figures 4B, 4C, 4E, and 4F). Quantitative analysis revealed that intratracheal administration of 1 mg CAT-expression plasmid formulated with the tetrafunctional block copolymer resulted in a level of CAT protein expression ranging from 0.01 to 315 ng in the different lung compartments, 48 h later (Figures 4E and 4F). Finally, addition of the total amount of CAT protein produced in the lungs upon tetrafunctional block copolymer gene transfer was 9.87 ng in pig n° 1402 (0.5 mg plasmid DNA, 48 h), 0.975 ng in pig n° 1411 (1 mg plasmid DNA, 24 h), 321.85 ng and 56.43 ng for pig n° 1403 and n° 1410, respectively (1 mg plasmid DNA, 48 h). Although the number of experimental subjects was limited, clear indications emerged. First, gene transfer measured 48 h after transfection appears to be much greater than that measured after 24 h. Second, the administration of 1 mg DNA appears to result in a higher level of gene transfer than administration of 0.5 mg DNA. However, the most striking observation was that liposomal-mediated gene transfer was far less efficient than tetrafunctional block copolymer-mediated gene transfer. Although limited in test subject numbers, our study indicated that this difference in efficacy was in the range of two orders of magnitude (Figure 5).

**Figure 6:**
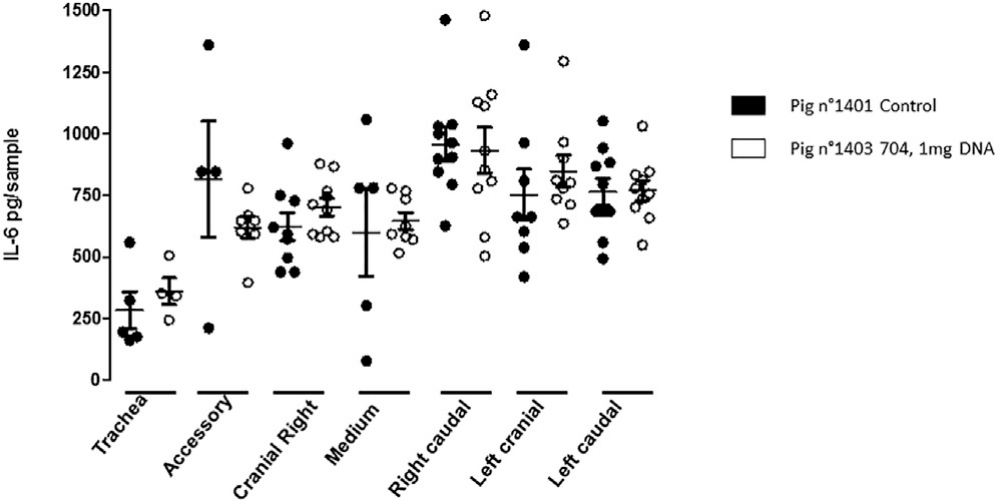
IL-6 Expression in Response to Tetrafunctional Block Copolymer-Mediated Gene Delivery of 1 mg Plasmid Lung IL-6 contents were determined by enzyme-linked immunosorbent assay in the different lung compartments after intratracheal instillation of saline buffer (pig n° 1401) or 1 mg plasmid DNA complexed with the tetrafunctional block copolymer (pig n° 1403).

We next measured inflammatory response via the analysis of interleukin-6 (IL-6) production as a cytokine marker of inflammation.^39^ Figure 6 shows IL-6 production in lung samples obtained 48 h after administration of saline buffer (pig n° 1401) or 1 mg plasmid DNA complexed with tetrafunctional block copolymer (pig n° 1403, in which the highest levels of transgene expression were detected; Figure 4D). Statistical analysis revealed a lack of difference between the two groups, suggesting a lack of inflammation, at least at this time point.

## Discussion

The validation in larger animal models of a proof of principle established in mice is an important step toward the utilization of novel therapeutics in patients. This is particularly the case for non-viral gene therapy, a field in which scalability can be hampered by problems of vector production and/or technical difficulties such as vector complexation and formulation stability. After a first series of experiments in mice,^35^, ^36^ we decided to validate the efficacy of tetrafunctional block copolymer-mediated lung gene transfer in newborn piglets. While previous validations of other non-viral lung gene delivery vectors have been performed using sheep,^13^, ^14^ our choice was guided by the availability of CF pigs^40^, ^41^ and by the fact that these animals recapitulate some anomalies found in young human CF patients.^42^ For example, newborn CF pigs develop air-trapping as a sign of airway obstruction before the appearance of airway infection, inflammation, and mucus obstruction.^43^ In this context, our study on wild-type newborn pigs is a step toward the validation of using tetrafunctional block copolymers as non-viral gene delivery vectors for *CF.*

Although the number of experimental animals was low, the results on newborn pigs confirm the conclusions obtained in mice and clearly indicate that tetrafunctional block copolymer-DNA complexes are more efficient gene-delivery vectors than liposomal-DNA complexes. In mice, the difference in efficiency was in the range of 3- to 5-fold. In newborn piglets, this difference appears to be much higher and certainly well above one order of magnitude, with no elevated levels of IL-6, as a marker of inflammation,^39^ measured 48 h after administration of the treatment. The lack of adverse reaction is also highlighted by the fact that the blood parameters measured before and after treatment were within the normal range (Table 1 and Chevaleyre et al.^44^). All together, these results are surprising in the light of data obtained using established cell lines. *In vitro*, cationic liposomes are well described as promoting gene^45^ and protein^46^ transfer. By contrast, block copolymers are very inefficient gene delivery vectors.^47^ The discrepancy in activity of non-viral gene transfer vectors in *in vitro* and *in vivo* situations has already been reported. For example, polypropylenimine dendrimer polypropylenimine dendrimers of third generation (PPIG3) -DNA nanoparticles are inefficient *in vitro* but very efficient at transfecting tumors *in vivo*.^48^, ^49^ These observations suggest that transfection *in vitro* and *in vivo* involve different mechanisms. Considering that most reagents are first selected *in vitro* before being tested *in vivo*, this hypothesis may explain why so many reagents are currently available for *in vitro* transfection, but so few are effective *in vivo*. Possible explanations for these differences are sparse,^47^ but some new concepts are starting to emerge. For example, *in vitro* and *in vivo* discrepancies in gene transfer in skeletal muscle can be unified by mechano-transduction.^50^ Further understanding these differences is likely to lead to the design of better non-viral gene transfer vectors *in vivo*.

In the present study, we used intratracheal instillations to deliver the gene-therapy vectors. This resulted in a non-homogeneous distribution of the gene transfer, with areas with high transgene activity next to areas not or poorly transfected. This effect is likely to be due to the fact that (1) the formulation was administered as a bolus and (2) the volume administered was relatively small. This type of unequal distribution of gene transfer in the lungs has already been reported in mice.^35^ In humans, lung administration of therapeutic compounds is performed using nebulizers. This type of device provides a level of comfort and ease of use for the patient. In addition, it allows a more homogeneous repartition of the aerosol. The majority of lung gene transfer studies using non-viral vectors have been performed using jet nebulizers.^51^, ^52^ In particular, this type of nebulizer is adapted to the delivery of DNA/liposome complexes.^52^ However, fundamental differences exist between the structures of DNA/liposomes and DNA/block copolymers. In the former, DNA is tightly bonded to liposomes, and the DNA is trapped in the structure.^53^, ^54^, ^55^ The DNA molecules are well protected and not very sensitive to shear forces exerted during the aerosolization process. As a result, the DNA/liposome retains its biological function.^52^ In the latter, the DNA molecules are exposed at the particle surface^24^ with weaker bonds to the block copolymers, leading to a situation in which the DNA is more susceptible to the type of degradation observed with naked plasmid DNA.^56^ The implementation of block copolymer-DNA complexes in lung gene therapy may therefore rely on the utilization of new types of nebulizers that can aerosolize a solution with reduced shear and cavitational stresses. A surface acoustic wave (SAW)-type nebulizer may be appropriate,^57^ although this type product is not currently commercially available. The SAW nebulizer has been shown to aerosolize naked plasmid DNA without noticeable degradation and to deliver naked plasmid DNA, leading to transgene expression in ovine lungs.^58^ In addition, when applied to rat lungs, plasmid DNA aerosolized using a SAW nebulizer retained its vaccinating potential.^58^

In conclusion, the present study highlights the potential of tetrafunctional block copolymers as non-viral vectors for lung gene therapy. Efforts should be focused on the availability of an aerosol device optimized for delivery of this formulation.

## Materials and Methods

### Animals

All experiments were conducted in accordance with EU guidelines and French regulations (DIRECTIVE 2010/63/EU, 2010; code rural, 2018; Décret n°2013-118, 2013). All experimental procedures were evaluated and approved by the Ministry of Higher Education and Research (notification APAFIS#5817-2016062316448313 v4). Pig procedures were evaluated by the ethics committee of the Val de Loire (CEEA VdL, committee number n° 19) and took place at INRA Experimental Infection Platform PFIE. A total of eight newborn large-white piglets (four females and 4four males, average weight 1.64 ± 0.13 kg) were used for the experiments. The piglets were allowed to suckle colostrum until intra-tracheal gene delivery and then hand-fed every 2 h. The physical condition of all animals was monitored twice per day. Animal welfare was determined by assessing the following parameters: general condition, feeding, body temperature, heart rate, respiratory rate, mucous color, feces, nasal discharge, coughing, and weight. Social and material enrichment was provided to maintain pig welfare.^44^ A protocol was set up as follows. Pigs would be euthanized when they showed at least three of the following major clinical signs: pain and distress, hyperthermia (>41°C), prostration, anorexia, diarrhea and/or vomiting, significant weight loss, tissue necrosis, biting. No animal became unexpectedly ill or died during the procedures.

### Plasmids

The plasmid pCIK-CAT (pCAT, 4.7 kb) encoding the *E. coli* CAT reporter gene (GenBank: NC_023277) was originally purchased from Bayou BioLabs (Harahan, LA, USA) and was a generous gift of D. Gill, Oxford, UK. It was purified from recombinant *E. coli* using the EndoFree plasmid purification kit (QIAGEN, Chatsworth, CA, USA). DNA integrity was monitored on a 0.8% agarose gel. Absence of endotoxins was verified by adding 1 mg plasmid DNA on to Caco-2 cells. Lactate deshydrogenase (LDH) and cytokine measurement in the culture medium was performed, as previously described,^59^ 24 h later to quantify the extent of cell death.

### Liposome

CholP was synthesized as previously described.^60^ DOPE and DMPE-PEG5000 were from Aveni-Polar (Alabaster, USA). CholP/DOPE (1/2, molar/molar [M/M]) liposomes were prepared by dissolving CholP and DOPE in chloroform at a molar ratio of 1. Chloroform was evaporated, and the dry lipidic film was hydrated with deionized water overnight at 4°C. A solution of DMPE-PEG5000 at 10 mg/mL in water was added to pre-formed CholP/DOPE liposomes at 20 mM positive charges to produce CholP/DOPE/DMPE-PEG5000 liposomes. DNA complexes with CholP/DOPE/DMPE-PEG5000 were prepared by mixing equal volumes of liposomes in water with DNA solution at 2 mg/mL in 300 mM NaCl. One milliliter of this solution was then administered to the piglet lung.

Mixing of plasmid DNA with various amounts of CHOLP liposomes led to particles of approximately 280 nm diameter. Dynamic light scattering analysis of DNA complexes^24^ showed that above a theoretical charge ratio of 2+/−, complexes were not colloidally stable as evidenced by a mean diameter above 600 nm. Ethidium bromide fluorescence measurements indicated that above a CholP liposomes-DNA charge of 2+/−, the fluorescence intensity was close to zero, indicating that all DNA molecules were complexed with CholP liposomes, preventing intercalation with ethidium bromide.

We investigated whether those aggregated colloidally unstable DNA complexes at a charge ratio of 2+/− could be protected from aggregation if a colloidal steric stabilizer was present in the solution. We synthesized CholP liposomes with the presence of various DMPE-PEG5000 and measured the colloidal stability when associated with a fixed amount of DNA. Dynamic light scattering analysis showed that for a DMPEPEG5000-DNA weight ratio of 2, DNA complexes displayed a mean diameter of 239 nm, while a mean diameter above 600 nm was observed with DMPE-PEG5000-DNA ratio below 2. Therefore, CholP/DOPE/DMPE-PEG5000-DNA complexes at a charge ratio of 2+/− and a DMPE-PEG5000-DNA ratio of 2 (w/w) were selected for further studies of piglet lung transfection.

### Tetrafunctional Block Copolymer 704

The tetrafunctional block copolymer 704 was provided by In-Cell-Art (Nantes, France). Stock solutions (20% w/v) were prepared in water and stored at 4°C. Formulations of DNA with copolymers were prepared by mixing equal volumes of the tetrafunctional block copolymer stock solution in water with plasmid DNA solution at the desired concentration in buffered solution.^61^ The physicochemical properties of the DNA/tetrafunctional block copolymer 704 complex used for *in vivo* experiments have been described previously.^35^

### Intratracheal Gene Delivery

Piglets were directly anesthetized with Vetflurane (Virbac, France). The trachea of each pig was intubated and ventilated with a Fabius Tiro Ventilator (Dräger, Telford, PA, USA). Ventilator settings were as follows: volume controlled mode, tidal volume = 8–10 mL.kg-1, positive end-expiratory pressure = 5 cm H_2_O, respiratory rate = 15 breath.min-1, inspiratory/expiratory ratio = 0.5, 50% oxygen.^44^ Piglets were inoculated with 1 mL of either PBS (control), DNA complexed with CholP/DOPE/DMPE-PEG5000, or the tetrafunctional block copolymer 704, into the carina using an esophageal probe. The pigs were ventilated mechanically until they recovered from anesthesia. The whole procedure lasted less than 20 min.

The piglets were sedated with intramuscular ketamine (20 mg/kg; Imalgene, Mérial, France) and xylazine (2 mg/kg; Rompun, Bayer, Germany) and then euthanized by intravenous injection of Doléthal (Vetoquinol, France, 250 mL, 50 mg/kg). The piglets’ lungs were dissected. The different lobes of the lungs (see Figure 1) were separated. Small cubes (around 1 cm^3^) were cut, placed in numbered Precellys tubes, and immediately frozen at −80°C. A numbering system allowing the identification of the lobe of origin of each sample was used.

### Hematological Analyses

Blood cells were counted with an MS9-5 Haematology Counter (digital automatic hematological analyzer, Melet Schloesing Laboratories, France^44^). Twenty-nine parameters were analyzed, which characterized three categories of blood cells: (1) total white blood cells (lymphocytes, monocytes, neutrophils, eosinophils, basophils, and other white cells), (2) red blood cells, and (3) platelets. The white blood cell and neutrophil counts were followed to monitor the inflammatory response after nebulization of the tetrafunctional block copolymer-DNA complex. The red blood cell parameters and platelet counts were followed to monitor the welfare of the newborn piglets.

### CAT Reporter Gene and IL-6 Assays

Tubes containing the samples were thawed for 5 min at room temperature and homogenized using a refrigerated Precellys tissue homogenizer. The tubes were then collected and centrifuged (10,000 × g, 10 min, 4°C). Supernatants were collected and used to measure transgene expression and IL-6 production. CAT expression was quantified as previously described.^23^ In control animals (n = 2), intratracheal administration of saline solution led to a level of CAT expression below the detection range of the ELISA in all samples tested (at least three per lung lobe, not shown). IL-6 levels were measured by ELISA using Abcam pig ELISA kits (Paris, France), according to the manufacturer’s instructions. For the determination of CAT protein content, an average of 81 samples were measured per lung. These were distributed as follows in the different compartments: trachea, 6; cranial right, 7; medium, 9; right caudal, 23; accessory, 6; left cranial, 9; and left caudal, 21. For IL-6 determination, the sample size for each compartment was (n control/n treated 704): trachea (5/4), right cranial (9/10), medium (5/8), right caudal (10/10), accessory (4/7), left cranial (8/9), and left caudal (10/10).

### Statistical Analysis

Statistical analyses were performed using Prism (GraphPad software).

## Author Contributions

Sample collection, I.C., C.C., C.B., and G.V.; Regulatory process, M.R., I.C., and G.V.; Pig husbandry and anesthesia, inoculation of vectors, M.R., C.B., J.P., and A.P.; Plasmid production and biochemical assays, O.H., J.F., R.R., B.M., and N.H.-V.; Chemical synthesis and analytical chemistry, B.P.; Conception of the experiments, redaction of the article, I.C., M.R., N.H.-V., B.P., and G.V.

## Conflicts of Interest

B.P. owns stock in In-Cell-Art, which commercializes tetrafunctional block copolymers.
